# Durable Control and Overall Survival Benefit with Focal Reirradiation in Cervical Cancer

**DOI:** 10.7759/cureus.399

**Published:** 2015-12-11

**Authors:** Arya Amini, Whitney Sumner, Christine M Fisher

**Affiliations:** 1 Department of Radiation Oncology, University of Colorado School of Medicine

**Keywords:** cervical cancer, recurrence, reirradiation, local control, lymph node recurrence, intensity-modulated radiotherapy, imrt, non-central

## Abstract

Local recurrence following definitive chemoradiation in cervical cancer ranges from 5-18%. Currently, there are limited options available for patients recurring in para-aortic or iliac lymph nodes. We present two cases of reirradiation for non-central recurrences with intensity-modulated radiotherapy (IMRT). Both patients achieved a complete response with manageable short-term toxicities and no late adverse effects with a follow-up of over one year and three years after reirradiation. While further follow-up is needed, IMRT is a feasible and safe option in cervical cancer patients with recurrent in-field failures involving the lymph nodes.

## Introduction

There are 527,600 new cases of cervical cancer worldwide with an estimated 265,700 deaths [1]. Concurrent chemoradiation is the current standard of care for advanced stage cervical cancer and local recurrence following definitive treatment ranges from 5-18% [2]. There are limited treatment options for patients with recurrent or metastatic cervical cancer, and survival rates are dismal with an average median overall survival approximating one year [3]. Few studies, to our knowledge, have evaluated the successful use of intensity-modulated radiotherapy (IMRT) in the setting of localized recurrent cervical cancer in the lymph nodes.

## Case presentation

Informed patient consent was obtained from both patients.

### Case 1

**Figure 1:**
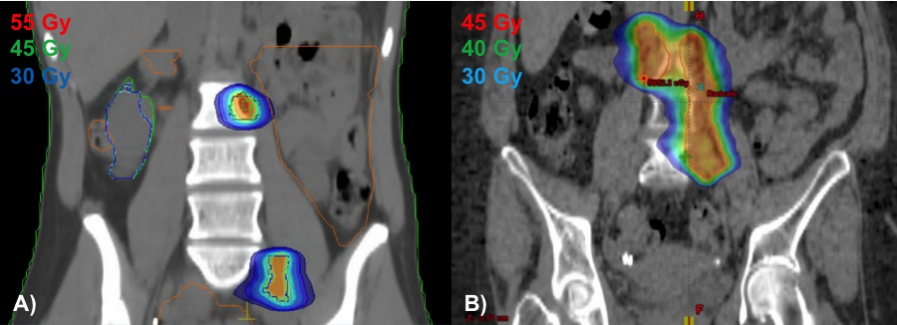
Coronal images of reirradiation treatment plans for Case 1 (A) and Case 2 (B)

Our first case is a 28-year-old female who initially presented in October 2012 with post-coital bleeding. Colposcopy identified a friable 2 cm mass on the cervix. She underwent a loop electrosurgical excision procedure (LEEP), which revealed a Grade 2 invasive adenocarcinoma with 1 cm stromal invasion, International Federation of Gynecology and Obstetrics (FIGO) 1B1. Positron emission tomography-computed tomography (PET-CT) revealed multiple fluorodeoxyglucose (FDG)-avid lymph nodes along the para-aortic and iliac vessels. Subsequent lymph node sampling confirmed pathologic involvement, American Joint Committee on Cancer (AJCC) Stage IVB, T1b1 N1 M1. She then received 50 Gy in 28 daily fractions to an extended field with IMRT, plus a boost to grossly positive nodes to 9 Gy in five fractions, and intracavitary tandem and ovoid brachytherapy to 30 Gy in five fractions with concurrent weekly cisplatin 40 mg/m^2^. RT was followed by four cycles of adjuvant cisplatin, which she completed in June 2013. Follow-up PET-CT demonstrated a complete response until February 2014 at which time there was noted to be an increased FDG uptake in at least three lymph nodes: one left para-aortic and two left external iliac nodes. Fine-needle aspiration (FNA) of the left para-aortic node was positive for rare, viable carcinoma cells in the background of extensive necrosis. She was started on bevacizumab, docetaxel, and topotecan. After completing six cycles, a repeat PET-CT demonstrated a complete response in one of the two left external iliac lymph nodes and a partial response in the left para-aortic node. Her case was discussed at Tumor Board with the decision to treat with consolidative RT for curative intent to the solitary para-aortic and external iliac node. She was treated with IMRT (45 Gy in 30 fractions twice daily) without chemotherapy, completing retreatment in October 2014 (Figure 1A). Acute side effects from RT included mild fatigue and increasing non-bloody bowel movements controlled with Imodium as needed. Following completion of RT, she was continued on maintenance bevacizumab. Repeat exam and imaging in November 2015 showed no evidence of disease, and at her last follow-up, her bowel movements had returned to baseline with no new late treatment-related toxicities (> one-year post-treatment).

### Case 2

The second case is a 53-year-old female who presented in July 2011 with vaginal spotting and discharge for three months. Her Pap test revealed a high-grade squamous intraepithelial lesion (HSIL), staining for high-risk human papillomavirus (HPV). Physical exam identified a 6 x 8 cm mass without parametrial involvement, and biopsy returned positive for invasive squamous cell carcinoma, FIGO 1B2. PET-CT also noted several prominent para-aortic and iliac lymph nodes: AJCC Stage IVB, T1b2 N1 M1. She underwent IMRT utilizing a simultaneous integrated boost technique to a dose of 52.5 Gy in 25 daily fractions to gross disease, 45 Gy in 25 fractions to pelvic nodes, and a boost of 4.2 Gy in two fractions to a residual, enlarged left external iliac node, delivered with weekly cisplatin 40 mg/m^2^. Additionally, she received intracavitary tandem and ovoid brachytherapy to 28 Gy in four fractions. She completed RT in October 2011. She tolerated treatment well with occasional nausea, vomiting, and diarrhea controlled with Lomotil and Imodium as needed. A subsequent PET-CT in September 2012, unfortunately, demonstrated an increased FDG uptake in multiple lower para-aortic nodes. Her case was discussed in Tumor Board with the decision to proceed with reirradiation for curative intent. She then received 45 Gy in 30 twice daily fractions with IMRT to the involved para-aortic lymph nodes without chemotherapy, completing treatment in November 2012 (Figure 1B). She tolerated treatment well with increasing loose stools managed with Imodium prn. On her most recent exam and follow-up imaging in September 2015 (three years post-retreatment), she had no evidence of disease and no long-term side effects.

## Discussion

Recurrence in the iliac and para-aortic lymph nodes often occurs following definitive treatment for cervical cancer [4]. The two cases presented in this report demonstrate a remarkable treatment response to IMRT in the setting of in-field lymph node recurrence treated for curative intent. With a follow-up of over one-year and three years, both patients achieved a complete response to therapy with no late side effects to date. To our knowledge, there is very little reported on reirradiation with IMRT for recurrent cervical cancer involving pelvic or abdominal lymph nodes. Single-institution studies and case reports have reported outcomes of reirradiation with brachytherapy for localized cervical cancer [5]. Interstitial brachytherapy can provide excellent definitive local control for recurrent or residual carcinoma of the cervix. However, in the setting of our two cases, where the site of recurrence was not localized to the cervix, brachytherapy would not have been feasible. Additionally, while stereotactic body radiotherapy (SBRT) would also be a potential option, data using reirradiation with hyperfractionation (twice daily RT) in other cancer sites have demonstrated excellent local control rates with tolerable side effects, given its biology for potentially improved normal tissue toxicity [6].

The concern for reirradiation with EBRT stems from the potential toxicities in normal tissues, such as small bowel, which can lead to short- and long-term side effects, including strictures, bowel obstruction, and tissue necrosis. To reduce surrounding normal tissue doses and overall short-term and long-term side effects from treatment, radiation oncologists have transitioned away from conventional external beam plans with large treatment fields and are now treating with more conformal plans, especially in the reirradiation setting. Much of this is made possible by IMRT, which has become increasingly utilized [7]. Additional technologies are also becoming incorporated in the field. For example, one case report evaluated the use of carbon ion RT in the setting of lymph node recurrence of cervical cancer near the border of the prior RT field; two patients were presented in the study, retreated to 48 Gy in 12 daily fractions with carbon ions, and achieving a complete response with no late adverse effects [8]. Presently, carbon ion treatment is not available in the United States, but results are promising.

## Conclusions

The management of cervical cancer using modern day techniques, including IMRT, have substantially improved conformity and reduced dose to organs at risk. Here, we provide two successful cases of reirradiation using IMRT in the setting of lymph node recurrence in cervical cancer. While further follow-up is needed, IMRT is a feasible and safe option in cervical cancer patients with in-field recurrences involving the lymph nodes.
